# A Novel Role for a Major Component of the Vitamin D Axis: Vitamin D Binding Protein-Derived Macrophage Activating Factor Induces Human Breast Cancer Cell Apoptosis through Stimulation of Macrophages

**DOI:** 10.3390/nu5072577

**Published:** 2013-07-08

**Authors:** Lynda Thyer, Emma Ward, Rodney Smith, Maria Giulia Fiore, Stefano Magherini, Jacopo J. V. Branca, Gabriele Morucci, Massimo Gulisano, Marco Ruggiero, Stefania Pacini

**Affiliations:** 1 Macro Innovations Ltd., CB4 0DS Cambridge, UK; E-Mails: youcanfindlyn@hotmail.co.uk (L.T.); eward@macroinnovations.co.uk (E.W.); rsmith@macroinnovations.co.uk (R.S.); 2 Department of Experimental and Clinical Biomedical Sciences, University of Firenze, 50134 Firenze, Italy; E-Mail: giulietz@hotmail.it; 3 Department of Experimental and Clinical Medicine, University of Firenze, 50134 Firenze, Italy; E-Mails: stemaghe@libero.it (S.M.); jacopo.branca@libero.it (J.J.V.B.); gabriele.morucci@unifi.it (G.M.); massimo.gulisano@unifi.it (M.G.); stefania.pacini@unifi.it (S.P.)

**Keywords:** vitamin D, macrophages, breast cancer, human, apoptosis

## Abstract

The role of vitamin D in maintaining health appears greater than originally thought, and the concept of the vitamin D axis underlines the complexity of the biological events controlled by biologically active vitamin D (1,25(OH)(2)D3), its two binding proteins that are the vitamin D receptor (VDR) and the vitamin D-binding protein-derived macrophage activating factor (GcMAF). In this study we demonstrate that GcMAF stimulates macrophages, which in turn attack human breast cancer cells, induce their apoptosis and eventually phagocytize them. These results are consistent with the observation that macrophages infiltrated implanted tumors in mice after GcMAF injections. In addition, we hypothesize that the last 23 hydrophobic amino acids of VDR, located at the inner part of the plasma membrane, interact with the first 23 hydrophobic amino acids of the GcMAF located at the external part of the plasma membrane. This al1ows 1,25(OH)(2)D3 and oleic acid to become sandwiched between the two vitamin D-binding proteins, thus postulating a novel molecular mode of interaction between GcMAF and VDR. Taken together, these results support and reinforce the hypothesis that GcMAF has multiple biological activities that could be responsible for its anti-cancer effects, possibly through molecular interaction with the VDR that in turn is responsible for a multitude of non-genomic as well as genomic effects.

## 1. Introduction

The so-called vitamin D axis is involved in various aspects of human breast cancer, the most common human tumor. The vitamin D axis is composed of the biologically active form of vitamin D (1,25(OH)(2)D3), and by two proteins that specifically bind it. These proteins are the vitamin D receptor (VDR) and the vitamin D binding protein that is the precursor of the vitamin D binding protein-derived macrophage activating factor, also termed GcMAF [1]. The role of vitamin D in human breast cancer is witnessed by the number of studies that have been published on the subject [2]. More intriguing, however, is the relative lack of information about GcMAF and human breast cancer; in fact, in the peer-reviewed literature, as of today (May 2013), there are only four studies on this subject. In two of these studies, the effects of GcMAF were observed on the human breast cancer cell line MCF-7 *in vitro* [3,4]. Another study examined the glycosylation status of vitamin D binding protein in cancer patients including breast cancer patients [5], whereas a less recent study reported the effects of administering GcMAF to metastatic breast cancer patients [6].

It is interesting to notice that no studies have, so far, been performed in order to assess whether GcMAF, which is a known powerful activator of macrophages, was indeed capable of activating macrophages that could in turn “attack” human breast cancer cells. There is indirect evidence suggesting that GcMAF activates macrophages that infiltrate experimental tumors in animal models [7,8]. This evidence, however, is indirect and, most important, refers to experimental tumors other than human breast cancer. In addition, since the observations quoted above were performed in experimental animals, the presence of confounding factors associated with the complexity of the responses of the whole organism to the presence of transplanted or advanced tumors, limits the possibility of interpretation of the presented results.

Therefore, in order to fill this gap of knowledge, we performed experiments to provide clear-cut evidence that GcMAF, as part of the vitamin D axis, activates normal macrophages that in turn exert a tumoricidal action against human breast cancer cells without the presence of confounding factors.

## 2. Experimental Section

Purified, activity-tested GcMAF was obtained from Immuno Biotech Ltd., Guernsey, Channel Islands. Paricalcitol was from Abbott, Roma, Italy. All other reagents were from Sigma Aldrich, Milano, Italy.

### 2.1. Cell Lines

**Figure 1:**
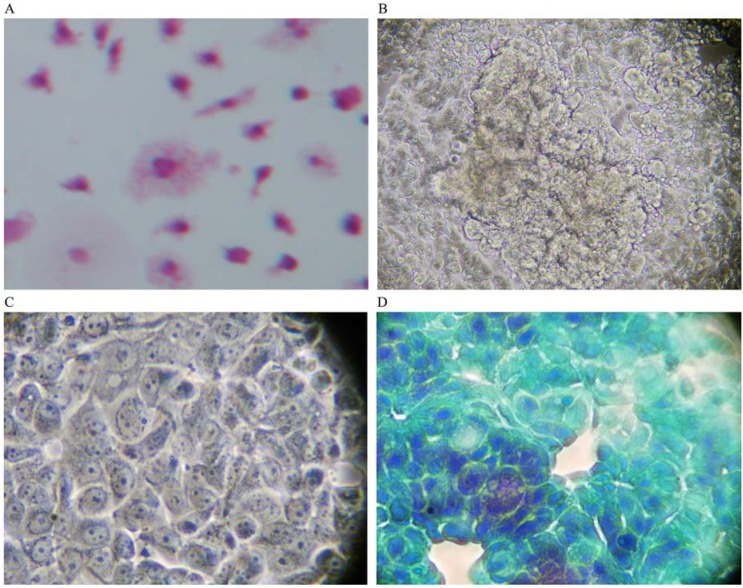
(**A**) Haematoxylin-eosin staining (magnification 300×); in the absence of GcMAF, small macrophages do not appear to interact with MCF-7 human breast cancer cells. The picture refers to 40 h co-culture. (**B**) Phase contrast microphotography (300×) of a cluster of cancer cells in the center. (**C**) At higher magnification (1200×) the cells appear densely packed. (**D**) Papanicolau staining (1200×); a cluster in the left lower side of the image. The nuclei are heavily stained and the perimeter of the cells is linear with no indents or signs of fragmentation.

**Figure 2:**
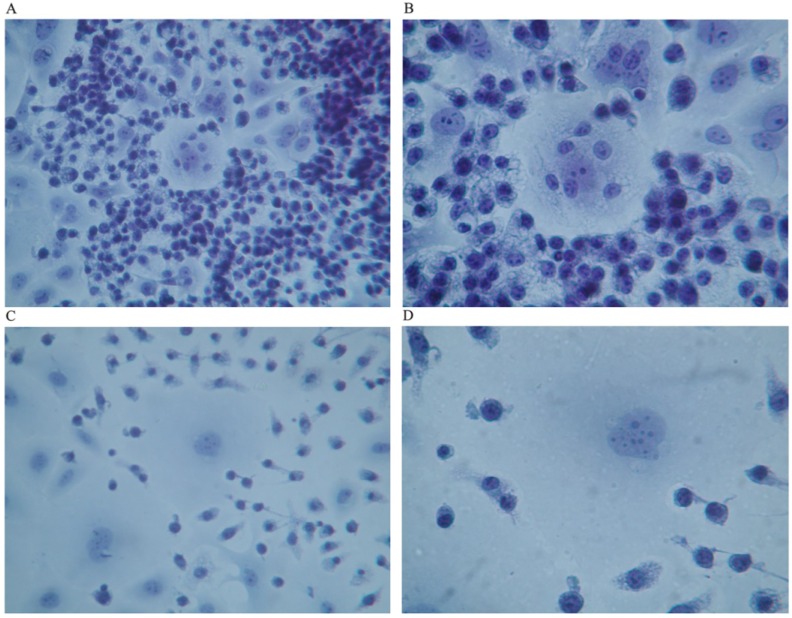
Co-culture of GcMAF-activated macrophages and human breast cancer cells; Papanicolau staining. (**A**) Cancer cells in the center are surrounded by hundreds of small macrophages (100×). (**B**) One human breast cancer cell is completely surrounded by macrophages that are also observable on top of the cell (200×). (**C**) Two large cancer cells are surrounded by GcMAF-activated macrophages (100×). (**D**) The same cell (200×); the chromatin in the nucleus is fragmented and, in the lower right corner, the cytoplasm is to be indented as if the two macrophages in that region were actively deconstructing the cytoplasm of the cancer cell.

Human breast cancer cells (cell line MCF-7) were obtained from the Istituto Zooprofilattico Sperimentale della Lombardia e dell’Emilia-Romagna, Brescia, Italy. Cells were routinely maintained at 37 °C in a humidified atmosphere of 5% CO_2_ in Eagle’s minimum essential medium in Earle’s Balanced salt solution, supplemented with 1 mM sodium pyruvate, 10% fetal bovine serum (FBS), 100 U/mL penicillin, and 100 µg/mL streptomycin (Invitrogen, Carlsbad, CA, USA). No 1,25(OH)(2)D3 was present in the culture medium. In experiments of co-cultures, macrophages (cell line Raw 264.7, HPA Culture Collection) were activated by culturing them in the same medium of MCF-7 cells and in the presence of 100 ng/mL GcMAF for 72 h prior to addition to the MCF-7 cell culture. GcMAF concentration was established by preliminary experiments showing a linear dose-response curve. The initial response was observed at 1 ng/mL and a plateau was reached at 100 ng/mL. These concentrations were consistent with the results previously reported [3,4]. Before addition to the MCF-7 cell culture, the macrophages were gently centrifuged and re-suspended in fresh medium in order to avoid transferring GcMAF to the co-culture. In this way, we could rule out direct effects of GcMAF on MCF-7 cells. The macrophages were added at a ratio of 1:1 to the MCF-7 cell culture. The cells were then allowed to settle for 1 h before time-lapse photography. Photography was taken over a 7-day period using an Olympus CK2 microscope and a GXCAM-3 with NCH Debut capture software. In the experiments described in Figure 1A, Figure 2, the cells were fixed and stained as described below 40 h after co-culturing them.

### 2.2. Study of Cell Morphology

Cell morphology was studied by phase-contrast microscopy using an Optika inverted microscope (Model XDS-2; Optika Microscopes, Bergamo, Italy). This microscope had a positive-phase plate for phase-contrast imaging below a long working distance condenser lens, and an 8 Mp digital camera with LCD Screen (Optika Microscopes, Bergamo, Italy). The light source was a 6 V/30 W halogen pre-centered illuminator, with adjustable intensity. Phase-contrast imaging was performed on living cells without any fixation or treatment. A series of digital images of living cells were recorded for each experimental point and the most representative were chosen.

Haematoxylin-eosin and Papanicolaou staining were also performed. This last staining results in very transparent cells, such that even thicker specimens with overlapping cells could be recorded. Briefly, cells were stained with Harris haematoxylin as nuclear stain. Orange G and EA-65 (Light Green, Bismarck Brown, and Eosin) were used for cytoplasmic staining (Sigma Aldrich, Milano, Italy). Slides were mounted with permanent mounting medium and observed under light microscopy (Nikon Instruments SpA, Milano, Italy). Pictures shown are representative of typical experimental data. Each experiment was performed with quadrupled samples and was replicated three times.

### 2.3. Study of Cell Proliferation

Assessment of cell proliferation was determined by a Calbiochem Rapid Cell Proliferation Kit (Calbiochem, D.B.A., Milano, Italy) [9]. Each condition was replicated with quadrupled samples and each experiment was replicated three times. Differences between experimental values were evaluated by the Student’s *t*-test.

### 2.4. Study of Amino Acid Alignments and Functions

Analyses were carried out on the nucleotide and amino-acid sequences of the genes coding for vitamin D binding protein/GcMAF (isoform 1 precursor; gi|324021743|ref|NP_001191235.1) and VDR (gi|38511972|gb|AAH60832.1) in Homo sapiens. In reference to the protein alignments, three parameters have been taken into account:

1. sequence identity
2. sequence similarity
3. hydrophobic profile

These criteria were evaluated because they determine the quality of the alignments. In addition, we evaluated the functional value of the amino acids replaced, *i.e*., the importance that any divergence assumes within the sequence. The values obtained have allowed the scores to be added, rather than multiplied, in the global calculation of alignment scores. Information concerning the selected genes was obtained from the database at the University of California, Santa Cruz [10] referring to the latest published version of the human genome [11]. In particular, we used the table refGene, containing all gene coding and non-coding for proteins. In this way, it was possible to obtain detailed information on human genes, such as: chromosome, position of the start and the end of transcription, position of the start and the end of coding part, and the number and the positions of exons. The annotations for the genes were obtained using the algorithm liftOver [12].

The presence of conserved elements within the alignment was verified by using the information contained in the phastConsElements28way table of the UCSC database. This table contains the predictions of conserved elements produced by the phastCons program. The positions were reported on the alignment. All operations, from the search of genomic information to the creation of the alignments, were made using R Statistical Mathematical Software. Once the sequences were aligned, the columns of residues were taken into consideration. Any lined-up residue is to be considered implicitly related to evolution. The hydrophobic profile was obtained using software on the website [13]. Among the several systems that can be used for the calculation of the index of the amino acid sequence hydrophobicity/hydrophilicity, we selected the Kyte and Doolittle’s method [14]. The three-dimensional protein structures of vitamin D-binding protein and VDR were obtained through the use of the PDB archive [15]. Superposition between the two structures was possible through the use of the Swiss Pdb Viewer software [16]. The PDB archive contains information about experimentally-determined structures of proteins, nucleic acids, and complex assemblies. SwissPdb Viewer is an application that provides an interface allowing analysis of several proteins at the same time. The proteins can be superimposed in order to deduce structural alignments and compare their active sites or any other relevant parts. Amino acid mutations, H-bonds, angles, and distances between atoms are easy to obtain thanks to the intuitive graphic and menu interface.

## 3. Results

When co-cultured with human breast cancer cells in the absence of GcMAF, macrophages did not interact with human breast cancer cells and their characteristically irregular morphology was maintained (Figure 1A). Little or no vacuoles could be observed in macrophage cytoplasm, indirect evidence of a lack of activation. As described before, human breast cancer cells exhibited their typically non-homogeneous morphology, with some cells larger than other. The morphology of the cells was irregularly polygonal. As expected, human breast cancer cells tended to grow, one on top of the other, forming clusters that reflected the characteristic loss of contact inhibition. Figure 1B depicts phase contrast microphotography of a cluster of human breast cancer cells cultured in the absence of macrophages or any other addition. Cancer cells are visible as cords of cells growing in multi-layers in the center of the Figure. At higher magnification (Figure 1C), the cells appeared densely packed, with linear, non-fragmented, margins, and with a clearly recognizable organization of chromatin inside the nucleus, indicating a strong synthetic activity compatible with the high rate of proliferation of these cells. The nucleoli are clearly visible. Figure 1D, shows Papanicolau staining of only human breast cancer cells; a significant cluster can be observed in the left lower side of the image. The nuclei appear heavily stained as expected in growing cancer cells. The perimeter of the cells is linear with no indents or signs of fragmentation. Empty (white) areas in the well are also clearly observable. These represent naked areas of the plastic well that reflect the loss of adherence typical of cancer cells. Loss of adherence is a pre-requisite for cellular detachment, invasiveness, and metastatic potential.

**Figure 3:**
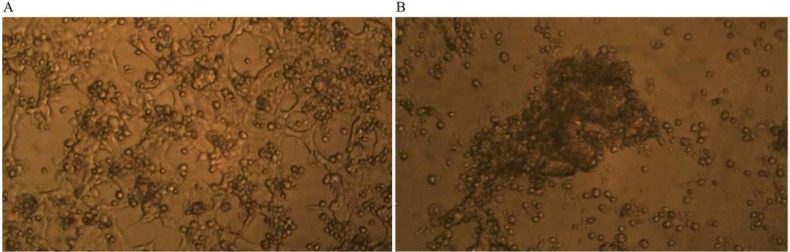
Phase contrast microphotography from time-lapse recording of co-culture of GcMAF-activated macrophages and human breast cancer cells. (**A**) Day one of co-culture; the cancer cells form an irregular layer. Individual cancer cells can be recognized. GcMAF-activated macrophages appear as small cells that are attached to the cancer cells, in most cases above them. (**B**) Day seven of co-culture. No individual cancer cell can be recognized. Their apoptotic bodies are grouped together in the center of the field, and most of the field is empty of cancer cells. Most GcMAF-activated macrophages surround and infiltrate the mass of cancer cell debris in the center.

However, when human breast cancer cells were co-cultured with macrophages that had been previously activated by GcMAF (100 ng/mL) for 72 h, the picture was completely different as shown in Figure 2, Figure 3. The pictures show co-culture of GcMAF-activated macrophages and human breast cancer cells after 40 h incubation. GcMAF-activated macrophages appeared as small cells that surrounded human breast cancer cells. Figure 2A (Papanicolau staining,) clearly shows a group of human breast cancer cell in the center of the image surrounded by hundreds of small macrophages. At higher magnification, (Figure 2B) one human breast cancer cell appears completely surrounded by macrophages that are also observable on top of the cell. The nucleus of the macrophages is well stained, whereas the chromatin in the nucleus of the cancer cell appears fragmented and disorganized. The nucleoli, however, are still recognizable; this phenomenon can be interpreted as an index of remaining synthetic activity as expected in cells undergoing active apoptosis. The cytoplasm of macrophages appears vacuolated thus suggesting active phagocytosis. Figure 2C shows another field where two large human breast cancer cells are surrounded by GcMAF-activated macrophages that appear to emit cytoplasmic extrusions that search for contact with the membrane of cancer cells. The cell in the center of Figure 2C, at higher magnification (Figure 2D), shows a peculiar aspect; the chromatin in the nucleus appears fragmented and, in the lower right corner, the cytoplasm appears to be indented as if the two macrophages in that region were actively deconstructing the cytoplasmic assembly of the cancer cell. A similar phenomenon can be observed on the left where two macrophages indent the cytoplasmic profile of the cancer cell.

It is worth noticing that all these morphological changes are consistent with the induction of apoptosis of human breast cancer cells by activated macrophages [17]. In particular, some of the morphological changes were consistent with the early phases of apoptosis and the morphology of the nucleus of human breast cancer cells shown in Figure 2 is almost superimposable to that represented in Figure 1 (left panel) of Hacker, 2000 [17]. Even the changes in the morphology of the cytoplasm were consistent with the induction of apoptosis by GcMAF-activated macrophages and the cytoplasm of human breast cancer cells showed the typical pattern of disintegration that precedes the formation of apoptotic bodies. In addition, in this case, the morphology of the cytoplasm of the cancer cells appears remarkably similar to that presented in Figure 1 (middle panel) of Hacker, 2000 [17]. Although the morphological features observed here are suggestive of active apoptosis, further studies using ELISA tests to quantify the level of human active caspase-3 protein, the major executioner protease in apoptosis, will determine quantitatively the degree of apoptosis induced by GcMAF-activated macrophages.

Time-lapse micro-photography shows that after about seven days of co-culture of GcMAF-activated macrophages with human breast cancer cells, the irregular growth of the breast carcinoma cells was arrested and the large protruding cell biomass was reduced. Figure 3A shows the human breast cancer cells and the GcMAF-activated macrophages at day one; the cancer cells, as expected, form an irregular layer that covers the field of observation. Individual cancer cells can be recognized as well as the naked areas of the plate as described above. GcMAF-activated macrophages appear as small cells that are attached to the cancer cells, in most cases, above them. It is interesting to notice that almost no macrophages can be observed in the naked areas of the plate, thus confirming the observation that GcMAF-activated macrophages seek for contact with the cancer cells. After seven days of co-incubation (Figure 3B), no individual cancer cell can be recognized. After macrophage-induced apoptosis, their apoptotic bodies are all grouped together in the center of the field of observation, and most of the field is empty of cancer cells. Most GcMAF-activated macrophages surround and infiltrate the mass of cancer cell debris in the center.

Taken together these results demonstrate for the first time that GcMAF-activated macrophages induce human breast cancer cell apoptosis and the subsequent reduction of the cancer cell mass following phagocytosis of apoptotic cancer cells by macrophages.

## 4. Discussion

It is long considered that the role of vitamin D in maintaining health is much greater than originally supposed, up to the point that some authors jokingly wonder whether “does vitamin D make the world go ‘round’?” [18]. The emergence of the concept of the vitamin D axis [1,19] further underlines the complexity of the biological events controlled by 1,25(OH)(2)D3 through its two binding proteins (VDR and vitamin D-binding protein/GcMAF) that interfere with a growing number of events at the cellular and molecular level. In this study we focused our attention on the product of deglycosylation of the vitamin D-binding protein that is GcMAF, probably one of the most potent macrophage activators so far discovered [20]. Our results demonstrate that GcMAF stimulates macrophages that in turn attack human breast cancer cells, possibly induce their apoptosis and eventually phagocytise them. These results are consistent with the observation that macrophages infiltrated experimental tumors implanted in severely immunodeficient mice after GcMAF injections [8]. However, at variance with the observation reported above, in our experiments we could rule out indirect effects due to the adaptive response of the whole organism to the presence of an advanced tumor and to the GcMAF-induced inhibition of angiogenesis with consequent tumor hypoxia and necrosis [8]. A limitation of the present study is represented by the use of only two cell lines, which are human breast cancer cell line MCF-7, and mouse Raw 264.7 macrophages. It should be noticed, however, that GcMAF exerted qualitatively superimposable effects on primary human mononuclear cells [21] and in the human monocytoid cell line, MonoMac 6 [22]. Future experiments will elucidate whether the effects observed in this study can be extrapolated to other human breast cancer cell lines challenged with GcMAF-activated human macrophages.

**Figure 4:**
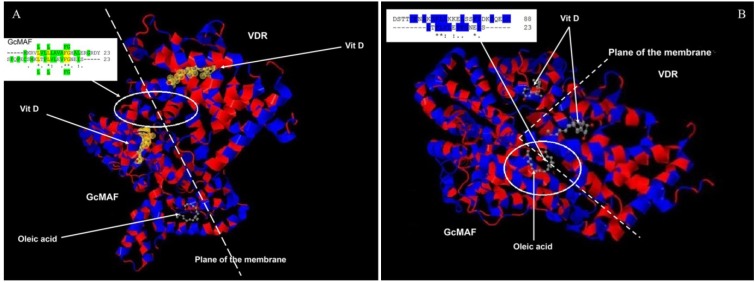
Amino acid alignments and three-dimensional protein structures of vitamin D-binding protein/GcMAF and VDR. (**A**) 23 hydrophobic amino acids of VDR (on the right), located at the inner part of the plasma membrane (dotted line), interact with 23 hydrophobic amino acids of the GcMAF (on the left of the Figure) located at the external part of the plasma membrane. In the insert the hydrophobic amino acids are highlighted in green and the four hydrophobic amino acids that are identical (L L FG) are highlighted in yellow and in green above and under the alignment. Vitamin D indicates 1,25(OH)(2)D3. (**B**) 23 hydrophobic amino acids of the VDR interact with a stretch of hydrophobic amino acids of the unsaturated fatty acid binding site of GcMAF. In the insert, eight amino acids with similar functional valence in a long stretch of hydrophobic amino acids highlighted in blue.

The observation that GcMAF, a component of the vitamin D axis, exerts tumoricidal effects on human breast cancer cells through macrophage activation raises the question of whether there is any interaction between GcMAF and the VDR. Such a type of interaction would be critical to understand the effects of 1,25(OH)(2)D3 and GcMAF at the molecular level. This question might appear odd at first, as, for many years, it had been thought that VDR was localized in the cytoplasm and in the nucleus, and GcMAF could not cross the plasma membrane and therefore had to be recognized by a surface receptor, possibly a lectin-type receptor [23]. However, the observation of an association between the polymorphisms of the gene coding for VDR, and differential responses to GcMAF in human monocytes [21], as well as with metastatic breast cancer [24], raises the apparently odd issue of a molecular interaction between GcMAF and the VDR. In support for this hypothesis there is the observation that the VDR translocates to the plasma membrane [25], and plasma membrane associated VDR is responsible for the rapid, non-genomic effects of vitamin D [26]. Thus, in order to verify the possibility of a molecular interaction between GcMAF and VDR, we compared the amino acid sequences corresponding to their respective 1,25(OH)(2)D3 binding sites. There are 23 hydrophobic amino acids near the amino terminus of GcMAF (-----MKRVLVLLLAVAFGHALERGRDY) and 23 amino acids near the carboxyl terminus of the VDR (SFQPECSMKLTPLVLEVFGNEIS-----). If these two sequences are aligned (Figure 4A), it is possible to observe, not only that in both proteins there is a long stretch [21,24] of hydrophobic amino acids (highlighted in green in Figure 4A, upper insert), but that four hydrophobic amino acids are identical (L L FG; indicated in yellow and in green above and under the alignment. The sequence of GcMAF is above). In addition, 11 amino acids have similar functional valence as indicated by the conventional symbols (*), (.) and (:). Therefore, in the 1,25(OH)(2)D3 binding domains of GcMAF and VDR there are in total 11 out of 23 amino acids that show functional identity or similarity and 13–14 that are hydrophobic. A molecular interaction between the two proteins can therefore be proposed (Figure 4A). According to this model, the last 23 hydrophobic amino acids of VDR (VDR is on the right of Figure 4A), located at the inner part of the plasma membrane (represented as a dotted line), could interact with the first 23 hydrophobic amino acids of the GcMAF (GcMAF is on the left of the Figure 4A) located at the external part of the plasma membrane, with 1,25(OH)(2)D3 (represented in yellow) sandwiched between the two vitamin D-binding proteins. Oleic acid, taken as an example of an unsaturated fatty acid bound to GcMAF [27], could stabilize the complex at the level of the plasma membrane. In fact, both 1,25(OH)(2)D3 and oleic acid in GcMAF are located in a shallow cleft of the GcMAF protein that makes them accessible to the plasma membrane. In addition to the mode of interaction proposed in Figure 4A, there could be further additional interaction that takes into consideration just the fact that vitamin D binding-protein (and therefore also GcMAF) binds unsaturated fatty acids as demonstrated by Williams *et al*., 1998 [27]. The fatty acid binding site is located between domains II and III, which is between positions 304 and 387. When we aligned the 23 hydrophobic amino acids of the VDR quoted above (represented in the insert in Figure 4B; also in this case, the sequence of GcMAF is represented above that of VDR) and the corresponding hydrophobic amino acids of the unsaturated fatty acid binding site of GcMAF (in particular, those in position 356–386), we observed that there was a significant degree of functional homology; in fact there are eight amino acids with similar functional valence in a long stretch of hydrophobic amino acids (highlighted in blue).

Therefore, it can be hypothesized that GcMAF and the VDR have multiple sites of interaction at the level of the plasma membrane. According to this model, the presence of 1,25(OH)(2)D3, in the culture medium should increase the effects of GcMAF by facilitating the interaction between GcMAF and VDR. Consistent with this model, we previously demonstrated that the effects of 1,25(OH)(2)D3 and GcMAF were synergistic in inhibiting MCF-7 cell proliferation [4], and the preliminary results reported in Table 1 indicate that GcMAF and paricalcitol, a non-hypercalcemic VDRagonist, also have synergistic effects. In the experiment described in Table 1, we chose to use paricalcitol instead of 1,25(OH)(2)D3 in order to determine whether the synergism between 1,25(OH)(2)D3 and GcMAF that we had previously observed [4], was to be ascribed exclusively to 1,25(OH)(2)D3, or could also be obtained with other VDR agonists. From the results presented in Table 1, it appears that paricalcitol, and, possibly, other VDR agonists, could fit the molecular model proposed in Figure 4.

**Table 1:** Effects of GcMAF and paricalcitol on Raw 264.7 macrophages. Raw 264.7 cells were incubated for 30 min with indicated additions. The effects of GcMAF on macrophage activation were assessed by determining cell proliferation. In fact, it was demonstrated that monocytes/macrophages activated by GcMAF administration immediately stop DNA replication and rapidly synthesize a large amount of Fc-receptors as well as an enormous variation of receptors [28]. Paricalcitol was added at the concentration of 300 fg/mL. At this concentration, paricalcitol did not exert any effect. In the presence of paricalcitol (300 fg/mL), the effect of 4 ng/mL GcMAF was identical to that of 40 ng/mL GcMAF in the absence of paricalcitol. These results demonstrate that the presence of a selective VDR agonist at a concentration that is not sufficient to activate VDR *per se* increases by an order of magnitude the response to GcMAF. Data are presented as means ± S.E.M. (*n* = 12). * *p* < 0.02 *vs.* control.

Treatment	Absorbance units (×10^3^)
Control (no addition)	390 ± 11
Paricalcitol	450 ± 10
GcMAF 40 ng/mL	379 ± 9 *
GcMAF 4 ng/mL + paricalcitol	327 ± 10 *

Taken together, these results support the hypothesis that the interaction between GcMAF and VDR might be facilitated by VDR agonists. This hypothesis is further strengthened by the recent observation that activated macrophages are able to generate enough biologically active vitamin D so as to be detectable in the general circulation [29], thus suggesting a paracrine/autocrine positive feedback loop.

## 5. Conclusions

The results presented in this study suggest that the role of vitamin D in physiology and pathology is far more complex than previously envisaged. Thus, in addition to 1,25(OH)(2)D3 itself, at least another component of the vitamin D axis, GcMAF, exerts significant effects at the cellular level and it appears that the effects of GcMAF are interconnected with VDR activation. Therefore, it can be hypothesized that these interconnections between 1,25(OH)(2)D3, GcMAF and VDR will be instrumental in devising new therapeutic usages for the components of the vitamin D axis.
